# Emodin Ameliorates the Efficacy of Carfilzomib in Multiple Myeloma Cells via Apoptosis and Autophagy

**DOI:** 10.3390/biomedicines10071638

**Published:** 2022-07-08

**Authors:** Chin-Mu Hsu, Chia-Hung Yen, Shu-Chen Wang, Yi-Chang Liu, Chien-Tzu Huang, Min-Hong Wang, Tzer-Ming Chuang, Ya-Lun Ke, Tsung-Jang Yeh, Yuh-Ching Gau, Jeng-Shiun Du, Hui-Ching Wang, Shih-Feng Cho, Yuhsin Tsai, Chi-En Hsiao, Samuel Yien Hsiao, Hui-Hua Hsiao

**Affiliations:** 1 Division of Hematology and Oncology, Department of Internal Medicine, Kaohsiung Medical University Hospital, Kaohsiung 807, Taiwan; e12013@gmail.com (C.-M.H.); ycliu@kmu.edu.tw (Y.-C.L.); gankay18@hotmail.com (C.-T.H.); dhlsy01128@gmail.com (M.-H.W.); benjer6@gmail.com (T.-M.C.); a9601082@gmail.com (Y.-L.K.); aw7719@gmail.com (T.-J.Y.); cheesecaketwin@gmail.com (Y.-C.G.); ashiun@gmail.com (J.-S.D.); joellewang66@gmail.com (H.-C.W.); sifong96@gmail.com (S.-F.C.); hsiao20191212@gmail.com (C.-E.H.); 2 Graduate Institute of Natural Products, College of Pharmacy, Kaohsiung Medical University, Kaohsiung 807, Taiwan; chyen@kmu.edu.tw; 3 Center for Cancer Research, Kaohsiung Medical University, Kaohsiung 807, Taiwan; 4 Department of Laboratory Medicine, Kaohsiung Medical University Hospital, Kaohsiung 807, Taiwan; sjwang@kmu.edu.tw; 5 Faculty of Medicine, College of Medicine, Kaohsiung Medical University, Kaohsiung 807, Taiwan; 6 Graduate Institute of Clinical Medicine, College of Medicine, Kaohsiung Medical University, Kaohsiung 807, Taiwan; 7 Graduate Institute of Chinese Medicine, School of Chinese Medicine, China Medical University, Taichung 404, Taiwan; yhtsai@mail.cmu.edu.tw; 8 Department of Biology, University of Rutgers-Camden, Camden, NJ 08102, USA; ucdsacnyu@gmail.com; 9 Cancer Center, Kaohsiung Medical University Hospital, Kaohsiung 807, Taiwan

**Keywords:** emodin, carfilzomib, reactive oxygen species (ROS), autophagy, apoptosis, p62, cell cycle

## Abstract

Background: Carfilzomib, the proteasome inhibitor, can increase the overall survival rate of multiple myeloma (MM) patients undergoing targeted therapy. However, relapse and toxicity present great challenges for such treatment, so an urgent need for effective combination therapy is necessary. Emodin is a natural chemical compound that inhibits the proliferation of various cancers and can effectively combine with other treatments. In this study, we evaluated the sensitizing effect of emodin combined with carfilzomib on MM cells. Methods: The cells were treated with emodin, carfilzomib, and a combination of drugs to determine their effects on cell proliferation and viability. The cell cycle distribution and reactive oxygen species (ROS) expression were measured by flow cytometry. The level of RNA and protein were analyzed through real-time qPCR and immunoblotting. Results: Emodin acted synergistically with carfilzomib to reduce the proliferation and viability of MM cell lines in vitro. Furthermore, the combination of emodin and carfilzomib increased ROS production, inducing apoptosis and autophagy pathways via caspase-3, PARP, p62, and LC3B. Conclusions: These results provide a molecular target for combination therapy in MM patients.

## 1. Introduction

**Figure 1:**
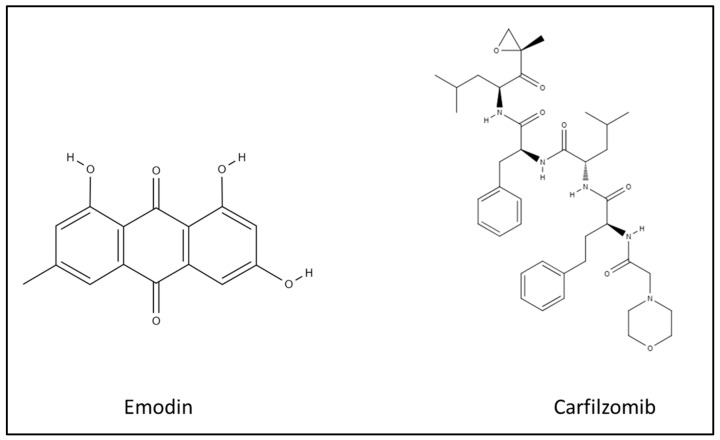
The structure of emodin and carfilzomib.

Multiple myeloma (MM) is incurable cancer characterized by clonal plasma cell proliferation with clinical anemia, reduced kidney function, bone destruction, and frequent infections. It accounts for approximately 10% of all blood cancers, with a five-year survival rate of 40%, and exhibits considerable variability in response to therapy [1]. Patients often experience repeated patterns of remission and relapse, but as the disease progresses, the period of remission becomes shorter, and the tumor cells become more aggressive, with most patients eventually dying from the refractory disease [2,3]. Over the past two decades, novel treatments, including proteasome inhibitors, have increased the median survival duration of MM patients; however, relapse and toxicity are major challenges for almost all MM patients [4,5,6]. Carfilzomib, a second-generation proteasome inhibitor, is used in the treatment of relapsed and refractory MM patients with less toxic side effects, but it is not more effective than the first-line bortezomib [7,8] (Figure 1). Carfilzomib can accumulate unfolding proteins, preventing degradation by inhibiting the β subunit of the proteasome and increasing intracellular oxidative stress to arrest the cell cycle and induce apoptosis and autophagy [9].

Emodin, a natural anthraquinone derivative, can inhibit the proliferation of cancer cells and has been shown to have therapeutic effects on many human malignant tumors, such as lung carcinoma, hepatoma, leukemia, and cervical cancer [10,11,12,13] (Figure 1). Emodin regulates the mechanism of anti-tumor proliferation via displaying the genotoxicity capacity and modulating signal transduction. Emodin can cause a DNA double-strand break by stabilizing the complexes of topoisomerase II-DNA and inhibiting ATP hydrolysis [14,15]. Haloemodin, derive from emodin, can also inhibit topoisomerase I and DNA gyrase [16]. Additionally, emodin also stimulates apoptosis by increasing the expression of the caspase genes and induces cell cycle arrest [10,11,12,13,17]. Emodin might behave as a Janus-activated kinase 2 inhibitor and have cytotoxic effects in MM cell lines, also inhibiting resistance and sensitizing the effect of chemotherapy, including gemcitabine, imatinib, and cisplatin, by inducing autophagy in cancer cells [18,19,20,21,22]. All these pathways are induced via reactive oxygen species (ROS), which can trigger apoptosis and autophagy through the activation of caspase genes and autophagy-related genes [23,24,25,26].

In the study, we examined whether the combination of emodin and carfilzomib is more effective against MM cells, showing that the combination treatment of emodin and carfilzomib was more growth inhibitory than either compound alone. The synergic efficiency of emodin and carfilzomib is via ROS-induced apoptosis and autophagy pathways.

## 2. Materials and Methods

### 2.1. Chemical Reagents

Emodin (Selleckchem, Houston, TX, USA) was dissolved in DMSO (dimethyl sulfoxide, Sigma-Aldrich, St. Louis, MO, USA) to a stock concentration of 10 mM and stored at −20 °C. Carfilzomib (Selleckchem, Houston, TX, USA) was dissolved in DMSO to a concentration of 100 μM. FBS (fetal bovine serum), RPMI1640 medium, L-glutamine, HEPES, sodium pyruvate, and Penicillin–Streptomycin were purchased from Gibco (Gibco, Thermo Fisher Scientific, Waltham, MA, USA). DMSO was purchased from Sigma-Aldrich (St. Louis, MO, USA).

### 2.2. Cell Culture

MM1S, RPMI8226, U266, and H929 (human MM cancer cell lines) were obtained from Bioresource Collection and Research Center (BCRC, Taiwan). Cells were cultured in RPMI-1640 supplemented with 10% FBS, 2 mM L-glutamine, 10 mM HEPES, 1 mM sodium pyruvate, and 1% Penicillin–Streptomycin in 5% CO_2_ at 37 °C. The cells were passed twice weekly.

### 2.3. Cell Viability

Cell viability was evaluated by the Alamar blue assay (DAL1025, Thermo Fisher Scientific, Waltham, MA, USA). Briefly, MM1S, RPMI8226, U266, and H929 cells were seeded into 96-well plates at a density of 20,000 cells per well and incubated in RPMI-1640 medium with 10% FBS in a final volume of 100 μL. After treatment with increasing concentrations of drugs for 72 h, 10 μL of Alamar blue was added to each well and incubated for 4 h. DMSO alone was used as a control. Absorbance was measured at 570 nm or 590 nm, and cell viability was expressed as a percentage of the control cells and the drug concentration.

### 2.4. Cell Cycle Analysis

MM1S, U266, and H929 cells were seeded into a 10 cm petri dish in medium containing 10% FBS without or with emodin (30 μM), carfilzomib (5 nM), separately or in combination (30 μM emodin and 5 nM carfilzomib). After 24 h, the MM cells were washed with cold PBS and fixed overnight in 75% ethanol at 4 °C as described previously [27]. The MM cells were washed with PBS, then incubated for 30 min at RT in the dark with a solution of 5 μg/mL propidium iodide (Sigma-Aldrich, St. Louis, MO, USA), 1 mg/mL RNase (Sigma-Aldrich, St. Louis, MO, USA), and 0.1% Nonidet P-40 (Sigma-Aldrich, St. Louis, MO, USA) before analysis using an LSR II Flow Cytometer. The intensity of PI fluorescence of individual nuclei was determined (at least 20,000 events were measured), and the data were analyzed via FlowJo software (FlowJo v10, BD Biosciences, San Jose, CA) to determine the percentage of cells at each phase of the cell cycle (sub-G0, G0/G1, S, and G2/M).

### 2.5. Measurement of Intracellular ROS

Intracellular ROS production was measured using 2′,7′ dichlorodihydrofluorescein diacetate (DCFH-DA, Cat.287810, Merck Millipore, Darmstadt, Germany). The cells were seeded into 6-well plates at a density of 1 × 10^5^ cells. The cells were treated with 30 μM emodin with or without 5 nM carfilzomib for 24 h in the 37 °C incubator with 5% CO_2_. The cells were washed with PBS twice and incubated with 1 mL of serum-free medium containing 500 nM of DCFH-DA. After 1 h of incubation at 37 °C, the cells were washed twice by PBS before the images were captured by flow cytometry. Fluorescence images were analyzed using FlowJo software, while fluorescence intensities in treated groups were expressed as normalized values of the control.

### 2.6. Quantitative Real-Time PCR

Total RNA was extracted using TOOLSmart RNA Extractor reagent (Tools, Taiwan) and reverse transcribed using a High-Capacity cDNA Reverse Transcription Kit (Thermo Fisher Scientific, Waltham, MA, USA). Each 20 μL reaction contained 2 × SYBR qPCR Master Mix (Thermo Fisher Scientific, Waltham, MA, USA), 1 μM forward and reverse primers 1 μL (Table 1), and 2 μL cDNA. The PCR was performed on the Applied Biosystems 7500 Real-Time PCR System, and the cycling conditions were 95 °C for 1 min, followed by 40 cycles of 95 °C for 10 s and 60 °C for 60. Target gene expression was quantified by the 2^−ΔΔCT^ method with GAPDH used as the internal control. The PCR reaction was repeated triplicate for each sample.

### 2.7. Immunoblotting

MM cells were treated with either emodin (30 μM), carfilzomib (5 nM), or a combination of both for 24 h, then washed twice with ice-cold PBS. Proteins were isolated from cell lysates using RIPA lysis buffer (Thermo Fisher Scientific, Waltham, MA, USA) containing 50 mM Tris–HCl, pH 7.5, 150 mM NaCl, 0.5% sodium deoxycholate, 1% NP-40, 0.1% SDS, and a protease inhibitor cocktail (Cyrusbio, New Taipei, Taiwan) and the protein concentration was determined by the Bradford assay (Bio-Rad, Contra Costa County, CA, USA). Thirty μg protein was separated by 10% SDS–PAGE, then immunoblotted using the following antibodies: PARP (GeneTex, Hsinchu, Taiwan), Caspase-3 (GeneTex, Hsinchu, Taiwan), LC3B (Cell Signaling Technology, Topsfield, MA, USA), and p62 (Cell Signaling Technology, Topsfield, MA, USA). GAPDH (Cell Signaling Technology, Topsfield, MA, USA) and β-actin (Cell Signaling Technology, Topsfield, MA, USA) were used as controls.

### 2.8. Statistical Analysis

All data were expressed as mean ± SD and analyzed using Student’s *t*-test or one-way ANOVA. A *p* value < 0.05 was considered statistically significant.

## 3. Results

### 3.1. Emodin Sensitizes Carfilzomib-Induced Death in MM Cell Lines

**Figure 2:**
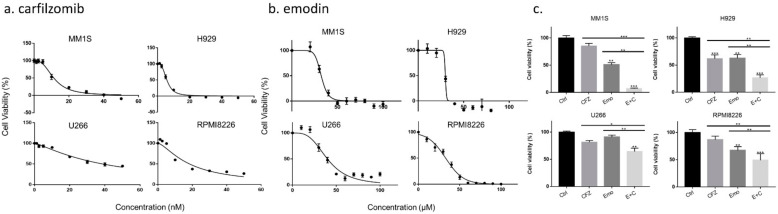
Cell viability of emodin (Emo), carfilzomib (CFZ), and combination drug of emodin and carfilzomib (E + C). (**a**) MM1S, H929, U266, and RPMI8226 cells were treated with various concentrations of CFZ for 72 h. (**b**) MM cells were treated with different emo concentrations for 72 h. (**c**) Emo (30 μM) enhanced the CFZ (5 nM) induced cytotoxicity to MM cells. * *p* < 0.05; ** *p* < 0.01; *** *p* < 0.001.

Carfilzomib inhibited the growth of MM1S, H929, U266, and RPMI8266 cells after 72 h treatment with IC50 values of 11.33 nM, 4.93 nM, 38.75 nM, and 18.25 nM, respectively (Figure 2a). Emodin also dose-dependently inhibited cell viability in the four MM cell lines, with IC50 values at 72 h of 31.29 μM in MM1S cells, 29.06 μM in H929 cells, 38.09 μM U266 cells, and 32.16 μM in RPMI8226 cells (Figure 2b). In the results, emodin, as well as carfilzomib, induced cell death in four MM cell lines after 72 h. Importantly, emodin and carfilzomib strengthened cell death when they were administered together (Figure 2c). The combination of emodin and carfilzomib further decreased the percentage viability compared to cells treated with either emodin or carfilzomib alone (*p* < 0.05), suggesting that emodin sensitizes myeloma cells to carfilzomib-induced cell death.

### 3.2. Emodin and Carfilzomib Modulated Cell Cycles of MM Cells Differentially

**Figure 3:**
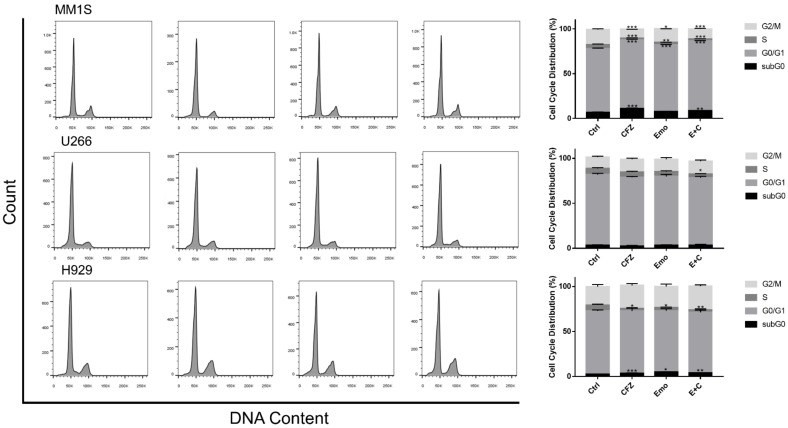
Differential cell cycle arrest with carfilzomib (CFZ), emodin (Emo) or combination drug of emodin and carfilzomib (E + C) for 24 h in MM cells. * *p* < 0.05; ** *p* < 0.01; *** *p* < 0.001.

Emodin and carfilzomib decreased the number of cells in the S phase of the three MM cell lines after 24 h (*p* < 0.05) (Figure 3). For MM1S and H929, the combination of emodin and carfilzomib increased the cell count in the sub-G0 phase (*p* < 0.05). Interestingly, the MM1S cells presented a trend of increased cell numbers in the G0/G1 phase and reduced cells in the G2/M phase; however, the H929 cells were increased in the G2/M phase. When taken together, these results suggest that emodin, carfilzomib, and the emodin/carfilzomib combination differentially sensitizes MM cells to induce cell cycle arrest.

### 3.3. Emodin and Carfilzomib Combination Increased ROS Production in MM Cells

**Figure 4:**
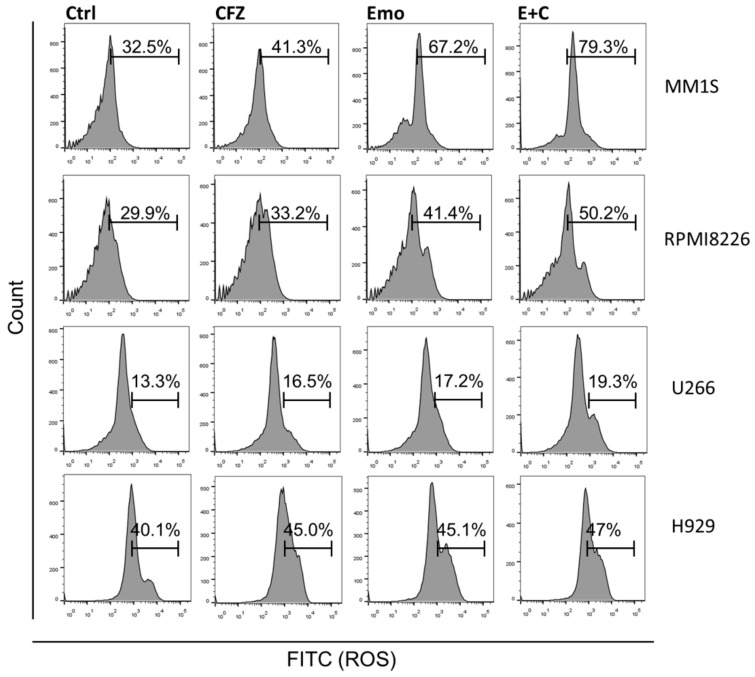
Carfilzomib (CFZ), emodin (Emo), or combination drug of emodin and carfilzomib (E + C) increased ROS production in MM cells.

In order to clarify whether oxidative stress is responsible for emodin/carfilzomib-induced ROS, MM cells were treated with emodin, carfilzomib, or a combination of both. Emodin and carfilzomib treatment increased ROS production in MM cells compared to the control (Figure 4). A combination of emodin and carfilzomib also enhanced ROS production in MM cells; thus, emodin and carfilzomib alone or a combination of both can increase the level of ROS in MM cells.

### 3.4. Emodin Ameliorates Stress-Associated Apoptosis and Autophagy Pathways after Combination with Carfilzomib

**Figure 5:**
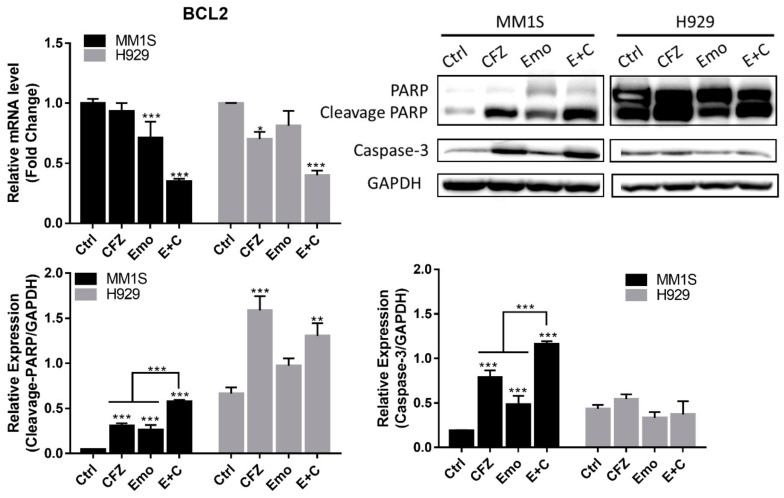
The combination therapy of carfilzomib (CFZ) and emodin (Emo) causes apoptosis in MM cells. The cells were incubated with CFZ (5 nM), Emo (30 μM), or the combination (E + C) for 24 h. * *p* < 0.05; ** *p* < 0.01; *** *p* < 0.001.

**Figure 6:**
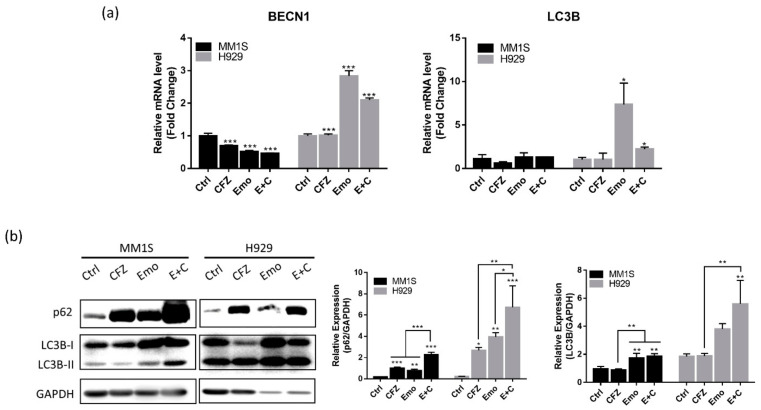
Carfilzomib (CFZ) and emodin (Emo) combination treatment modulated autophagy pathway. CFZ (5 nM), Emo (30 μM) and combination (E + C) treated MM1S and H929 for 24 h. (**a**) Relative RNA expression in BECN1 and LC3A; (**b**) The protein level of p62 and LC3B were detected by immunoblotting. * *p* < 0.05; ** *p* < 0.01; *** *p* < 0.001.

The exposure of MM1S and H929 cells to emodin (30 μM), carfilzomib (5 nM), or a combination of both inhibited BCL2 mRNA expression (*p* < 0.001), which was accompanied by significant caspase-3 activation and PARP cleavage after 24 h treatment, indicating that MM cells are undergoing apoptosis (Figure 5). The combination of emodin and carfilzomib enhanced apoptosis by increasing the protein expression of caspase and PARP cleavage compared to emodin or carfilzomib alone in MM1S (*p* < 0.001) (Figure 5). Regarding autophagy-associated genes, emodin, carfilzomib, and emodin/carfilzomib-treated cells expressed p62 and LC3B compared to the normal control (*p* < 0.05) (Figure 6). qPCR analysis also showed emodin but not carfilzomib-induced BECN1 and LC3A expression. The combination of both drugs also caused the expression of autophagy-associated genes, BECN1 and LC3A, in the MM cells.

## 4. Discussion

Carfilzomib is a second-generation proteasome inhibitor therapeutically effective in relapsed/refractory MM; however, it is associated with drug resistance and severe toxicity [28,29,30]. Emodin is the quinone derivative extracted from plants that can inhibit the growth of a variety of cancers in vitro and in vivo. Muto et al. demonstrated that emodin inhibits the proliferation of MM cells and induces apoptosis through the JAK2-STAT3 signaling pathway [18]. Zheng also reported that the emodin derivative E35 exhibits synergistic effects with bortezomib to induce MM cell apoptosis [19]. In the present study, emodin effectively reduced the survival rate of MM cell lines, with the combined use of emodin and carfilzomib having a synergistic effect (Figure 2c). Previously, it was demonstrated that emodin could enhance the effects of different chemotherapy drugs, inhibiting the growth of cancer cells [20,21,22,31]. Therefore, emodin and carfilzomib acted synergistically to compensate for the insufficiency of carfilzomib, sensitizing the cells to carfilzomib. Emodin is also used in combination with many chemotherapy drugs such as cisplatin, paclitaxel, taxol, and gemcitabine to enhance their effect, significantly inhibiting the growth of cancer cells.

The cell cycle is important for the regulation of cell proliferation and duplication. Emodin and carfilzomib alone could induce cell cycle arrest in MM cells at different phases. In previous studies, emodin was reported to increase the sub-G0 phase [17,32,33,34]. The present study revealed that all treatments reduced the number of cells in the S phase, with emodin and carfilzomib treatment arresting H929 cells in the G2/M phase and MM1S cells in the G0/G1. Emodin or carfilzomib can also induce both apoptosis and autophagy pathways.

ROS production is a common effect of chemotherapeutic drugs and is important for the induction of apoptosis and autophagy. In the present study, both emodin and carfilzomib generated ROS, with the combination of emodin and carfilzomib generating more ROS than either drug alone. Furthermore, emodin sensitized cells to carfilzomib to increase oxidative stress, producing more ROS, which indicates that ROS is important to induce cell death. The induction of oxidative stress is considered an important mechanism for proteasome inhibitors to induce death in MM cells [25,35,36,37]; therefore, it was hypothesized that emodin and carfilzomib increase ROS production to inhibit MM cells.

Regarding apoptosis, BCL2 mRNA decreased in cells exposed to emodin or carfilzomib, with the combination of both drugs further reduced BCL2 mRNA expression in MM cells. Immunoblotting showed significant changes in caspase 3 activity and cleavage PARP in response to emodin, carfilzomib, and combination treatment in MM1S especially (Figure 5). When taken together, these results indicated that emodin and carfilzomib could induce apoptosis, with the combination treatment enhancing apoptosis dependent on caspase activation and PARP cleavage in MM cells. Similar results were also found with resveratrol in combination with carfilzomib in multiple myeloma [38]. The transcription and translation levels of autophagy-related genes are not altered after low-dose carfilzomib treatment, but the combination of emodin and carfilzomib in MM cells significantly increased LC3B. The increased oxidative stress due to the combined carfilzomib and emodin treatment can activate autophagy through cellular p62 level alternation. ROS are known to induce both apoptosis and autophagy pathways in tumor cells by expressing genes such as caspase-3, PARP, and LC3B, thereby suppressing the malignant phenotype of tumor cells [39,40,41]; thus, emodin can sensitize cells to carfilzomib to induce apoptosis and autophagy.

## 5. Conclusions

Emodin increases carfilzomib-induced cytotoxicity of MM cells by increasing cellular ROS production to induce apoptosis and autophagy pathways. These results could provide a molecular targeting strategy for the treatment of MM.

## Figures and Tables

**Table 1:** The primer sequences of real-time qPCR.

Genes	Forward Primer	Reverse Primer
BCL2	CCTGTGGATGACTGAGTACCTGAAC	CAGCCAGGAGAAATCAAACAGA
BECN1	CTGGACACGAGTTTCAAGATCCT	GTTAGTCTCTTCCTCCTGGGTCTCT
GAPDH	GCACCACCAACTGCTTAGCA	TCTTCTGGGTGGCAGTGATG
LC3A	TCCTGGACAAGACCAAGTTTTTG	ACCATGCTGTGCTGGTTCAC

## Data Availability

Data is contained within the article.
